# Support for multiple classes of local expression clusters in *Drosophila melanogaster*, but no evidence for gene order conservation

**DOI:** 10.1186/gb-2011-12-3-r23

**Published:** 2011-03-17

**Authors:** Claudia C Weber, Laurence D Hurst

**Affiliations:** 1 Department of Biology and Biochemistry, University of Bath, Claverton Down, Bath, BA2 7AY, UK

## Abstract

**Background:**

Gene order in eukaryotic genomes is not random, with genes with similar expression profiles tending to cluster. In yeasts, the model taxon for gene order analysis, such syntenic clusters of non-homologous genes tend to be conserved over evolutionary time. Whether similar clusters show gene order conservation in other lineages is, however, undecided. Here, we examine this issue in *Drosophila melanogaster *using high-resolution chromosome rearrangement data.

**Results:**

We show that *D. melanogaster *has at least three classes of expression clusters: first, as observed in mammals, large clusters of functionally unrelated housekeeping genes; second, small clusters of functionally related highly co-expressed genes; and finally, as previously defined by Spellman and Rubin, larger domains of co-expressed but functionally unrelated genes. The latter are, however, not independent of the small co-expression clusters and likely reflect a methodological artifact. While the small co-expression and housekeeping/essential gene clusters resemble those observed in yeast, in contrast to yeast, we see no evidence that any of the three cluster types are preserved as synteny blocks. If anything, adjacent co-expressed genes are more likely to become rearranged than expected. Again in contrast to yeast, in *D. melanogaster*, gene pairs with short intergene distance or in divergent orientations tend to have higher rearrangement rates. These findings are consistent with co-expression being partly due to shared chromatin environment.

**Conclusions:**

We conclude that, while similar in terms of cluster types, gene order evolution has strikingly different patterns in yeasts and in *D. melanogaster*, although recombination is associated with gene order rearrangement in both.

## Background

In all well studied eukaryotic genomes gene order is known not to be random, with genes with similar expression profiles tending to cluster (see, for example, [1-4]). The model organisms used for work on gene order evolution are the yeasts, for which we have high-resolution gene order rearrangement data across a group of species, as well as excellent data on numerous additional parameters (for example, gene expression, and recombination rates) for one focal species, *Saccharomyces cerevisiae*. In *S. cerevisiae *we observe pairs or triplets of adjacent genes that are functionally related and very highly co-expressed [5-7]. Similarly, we find stretches of up to about 10 to 15 genes enriched for essential genes that also tend to be highly expressed [8]. We hereafter use the term 'cluster' to refer to neighborhoods of genes defined by local expression similarities, and the term 'co-expression' to refer to highly correlated expression patterns across multiple conditions or over a time course.

Do different types of clusters of similarly expressed genes behave as evolutionarily conserved units, or might the similar expression profiles merely be the result of transcriptional noise? In yeast, we see some evidence for the former possibility. In addition to the functional similarities observed in small co-expression clusters [6], both essential gene clusters and co-expression clusters show a tendency to be preserved as syntenic units over evolutionary time [8-11]. While genes that are in close proximity are also less likely to be rearranged, the above conservation of synteny cannot be accounted for by intergene distance (IGD) alone [9,11].

Thus, based on findings in yeast, it is tempting to speculate that eukaryotic genomes consist of stretches of genes with coordinated expression profiles that are maintained by natural selection. Can we be confident, however, that lessons from the model species have more general applicability? If not, we might need to consider genomes on a case-by-case basis. It is, for example, far from obvious that comparable clusters in other species also show gene order conservation, with reports being contradictory. Regions with a high density of essential genes are reportedly associated with increased regional linkage conservation in mice [12]. Similarly, early reports claimed a dearth of breakpoints in clusters of house-keeping genes [13] and conservation of small co-expressed clusters [14]. However, a more recent analysis [15] suggests, if anything, quite the opposite may be true, with highly co-expressed pairs being more likely to be rearranged. Likewise, functionally coordinated gene neighborhoods present in both humans and chimps are enriched for synteny breaks [16].

How might these findings be reconciled with what is observed in yeast? While conserved synteny is seen for the most highly co-expressed gene pairs in yeast, there are also many pairs showing moderate levels of co-expression but no functional similarity. This is likely to reflect noisy expression associated with the opening and closing of chromatin [17,18]. Similar broad scale noisy chromatin dynamics could explain why there are clusters of housekeeping genes. Given the high rate of rearrangement observed between highly co-expressed genes with short IGDs [15], it has been suggested that such noise driven co-expression may be disadvantageous.

One problem with prior analyses, outside of the yeasts, is a dearth of close comparator species, making genome-wide identification of breakpoints difficult. With the advent of the well-sequenced *Drosophila *genomes we can, however, now ask whether the lessons from our model species, the yeasts, also hold true within this group. Here, then, we consider the evolution and maintenance of clusters in *D. melanogaster*, making use of recent high-resolution data on the position of gene order rearrangements inferred from multiple sequenced *Drosophila *species [19].

Unfortunately, relatively little is known about whether the kinds of gene clusters described in other species are also present in *D. melanogaster*. The clusters described in other species appear to fall into two main categories: small co-expression clusters and large housekeeping/essential gene clusters. Small clusters of two to three genes that are highly co-expressed (assayed by Pearson's product moment correlation of expression values) and functionally coordinated (assayed by concordance of Gene Ontology (GO) or GO Slim categories) are seen in many species other than yeast, such as *Arabidopsis thaliana *[20] and, to a lesser extent, humans [6]. These we shall term type 1 clusters.

The second category of clusters of house-keeping [21] and/or highly expressed genes [22] in the human genome is likely to be the equivalent of (or closely related to) similarly sized clusters of the essential genes seen in both yeast and *Caenorhabditis elegans *[8,23]. In both of these species the clusters of essential genes also tend to have low recombination rates [8,23]. These larger clusters show little or no sign of co-expression and little or no sign of functional similarity [8,24]. We term these functionally uncoordinated clusters type 2 clusters. We shall assume, as seems defendable [24], that housekeeping clusters are the same as essential gene clusters.

Currently, it is not clear whether *D. melanogaster *has type 1 and/or type 2 clusters. That they have clusters of genes expressed in testes [25,26] and of immune genes involved in interactions with pathogens [27] suggests that they might well also have small type 1 clusters. We have previously shown that adjacent genes in *D. melanogaster *are more similar in terms of expression breadth than expected by chance [28]. This suggests that *D. melanogaster *may well have type 2 housekeeping clusters.

*D. melanogaster *is, however, unusual in having a third form of cluster that, to date, has not, to the best of our knowledge, been reported elsewhere. Spellman and Rubin [29] identified clusters that resembled type 1 clusters in showing co-expression, but resembled type 2 clusters in being large and having no functional similarity. This may simply reflect an inability to define functional co-ordination, but for want of evidence we shall consider the clusters observed by Spellman and Rubin as large but functionally uncoupled clusters that we name SR (for Spellman and Rubin) clusters.

Given the uncertainty over what kinds of cluster *D. melanogaster *has, we start by testing for the different forms of cluster. In addition to previously identified SR clusters, we provide evidence for small clusters of highly co-expressed genes and larger clusters of housekeeping genes. Given the evidence for small clusters, we also ask whether the SR clusters are biologically relevant units or whether they may reflect a methodological artifact. SR clusters were defined by considering all genes in a ten-gene window and asking whether the mean level of co-expression between them all was above some threshold. A given large 'cluster' could, however, actually contain, for example, two different small clusters that are uncorrelated with each other. While the strength of co-expression between the two clusters may be unremarkable, correlations within each of the unrelated clusters force the mean level over a threshold. Given the above scenario, it is far from clear that the large size of the cluster need be of any relevance and we may be better off considering the two smaller clusters in isolation. If such clusters are then grouped together, it might appear as though co-expression clusters have no functional significance even if each individual cluster is functionally coordinated. In principle, one cluster with very high co-expression scores could also push a ten-gene window over the threshold, the other genes in the window being irrelevant. To overcome this problem, we establish an algorithm whereby we define type 1 clusters by growing from a small co-expression cluster and extend only if the local genes are co-expressed with the core co-expressed set. We then consider the overlap between these co-expression clusters and SR clusters.

Finally, we ask whether the three cluster types are units of evolution, in the sense that they are domains of preserved synteny, as observed in yeast [8-11]. Consideration of rates of synteny preservation needs to control for background effects. In yeast, for example, two genes with only a small IGD between them are less likely to be rearranged [9,11]. Intergene distance is thus a potentially important covariate. Likewise, domains of high recombination rates tend to be domains of high-rearrangement rates [30,31]. Any preserved synteny thus may reflect covariance with the local recombination rates.

## Results

### Characterizing housekeeping clusters

#### *D. melanogaster *has clusters of housekeeping genes

We previously found that adjacent genes in *D. melanogaster *are more similar in terms of their tissue specificities than randomly selected genes [28]. To determine whether this might be due to low tissue specificity genes (that is, putative housekeeping genes) clustering, we asked whether broadly expressed genes tend to sit next to each other more often than expected by chance. We encoded low-specificity genes (tau ≤0.25) 1 and all other genes 0. For each set of neighbors along each chromosome, a switch was recorded for every transition between states as in [24]. For example, in a simple array of ten genes of which five are housekeeping and five not, maximum clustering would be found with the arrangement 1111100000. This has only one transition (between 1 and 0). By contrast, the less structured organization 0110010101 has seven state changes. The number of transitions between states in the real genome was lower than for each of 10,000 randomized sets where gene order was shuffled prior to recording the number of transitions (*P *< 9.999 × 10^-5^), except for chromosome 4 (*P *= 0.4326; median observed transitions 32, median expected transitions 32). Therefore, clustering of low tissue specificity genes exceeds random expectation, indicating that putative housekeeping genes in *D. melanogaster *cluster (with the exception of chromosome 4).

We next sought to exclude the possibility that this is accounted for by the presence of duplicates. Using all-against-all Blastp, all duplicate genes were detected using a cutoff value of e < 10^-7^, as in Spellman and Rubin's analysis, and one of each pair of duplicates was excluded (provided neither was already blacklisted). The transitions in the real genome still exceeded the simulations (*P *< 9.999 × 10^-5^), except for chromosome 4 (real median 32; simulated median 32; *P *= 0.4252). The same is also observed when genes detected in all of 14 adult tissues included in FlyAtlas [32] are encoded as 1 despite duplicate removal (for chromosome 4, *P *= 0.2134, real hits 27, median simulated hits 29; *P *< 9.999 × 10^-5 ^for all other chromosomes). Thus, we observe greater than expected clustering of putative housekeeping genes, defined as either low-specificity genes or genes expressed in all adult tissues.

We next defined clusters as stretches that begin and end with broad specificity genes (tau ≤0.25) and within which at least every fourth gene must be low specificity. Cluster span and number of low-specificity genes per cluster were recorded. We then filtered out all those clusters that could have occurred by chance by running 10,000 randomly shuffled genomes through the clustering algorithm. Chromosome 4, which did not show greater than expected local similarities in expression breadth, was excluded from the analysis. Only those clusters whose span and number of low-specificity genes had a <5% chance of occurring randomly were retained. We defined 512 clusters with a median of 5 genes and spanning, on average, 7 genes. Of these, 85 clusters with a median gene number of 9 and median span of 13 were found to have a <5% chance of occurring by chance. Maximum gene number was 40, and maximum span was 70.

We repeated the above, defining housekeeping genes as those genes expressed in all 14 adult tissues, and requiring housekeeping genes assigned to a cluster to be expressed in all tissues, allowing for jumps as above. We thus obtained fewer but slightly larger clusters. We detected 421 clusters with a median of 7 genes and span of 11, with 129 of these with a median of 12 genes and median span of 17 remaining after filtering. The maximum number of housekeeping genes found in a cluster was 57 with a maximum span of 86. Thus, as previously described in other organisms, *D. melanogaster *contains type 2 clusters of housekeeping genes. The former clusters defined by tau we refer to as tau clusters, and those defined with respect to the proportion of tissues showing evidence of expression we term breadth clusters, these both being housekeeping clusters. A compendium of all identified clusters and the genes therein is given in Additional file 1 (breadth clusters) and Additional file 2 (tau clusters).

### Housekeeping clusters do not show greater than expected functional coordination

Next we asked whether housekeeping clusters in *D. melanogaster *are functionally coordinated. Sixty-three of 88 housekeeping clusters (as defined by low tau) for which functional annotation was available show significant enrichment for at least one GO Slim term. This is more than expected based on 10,000 randomizations where clusters were populated with randomly selected genes and tested for enrichment (probability of observing as many or more enriched clusters = 0.0002). However, this could be owing to the presence of tandem duplicates, which are enriched for genes involved in developmental processes and transcriptional regulation [33]. When the test is repeated without duplicated genes, 51 clusters show a significant enrichment for at least one term, which is not significantly different from random expectation (*P *= 0.1201). Similar results are obtained when breadth clusters without duplicates are considered. The observed number of enriched clusters (68) is no greater than expected (*P *= 0.5275). Thus, as in yeast and *C. elegans*, large clusters of putative housekeeping or essential genes once filtered for duplicates do not appear to show functional coordination [8].

### The recombination rate in housekeeping clusters may be unusual

In yeast and worm, clusters of 'essential' genes (that is, knockout inviables) are conserved and have low recombination rates [8,23]. Similarly, there is a negative correlation between crossover rate and expression breadth in humans [34]. We therefore asked whether genes in housekeeping clusters tend to have lower recombination rates than the rest of the genome. Since chromosome 4 does not recombine nor show evidence of clustering, it was not considered in this analysis. When we consider recombination as estimated using the regression polynomial (RP) method [35], for tau clusters we indeed observe a reduced recombination rate (median cluster rate = 2.804; overall median rate = 2.934; Wilcoxon test *P *= 0.0006). However, no relationship is observed when we consider breadth clusters (*P *= 0.6421). These clusters could include some genes that are expressed at a low level due to leaky transcription, and could therefore be presumed to be a less suitable measure for identifying housekeeping clusters. Tau, on the other hand, takes into account how highly expressed a gene is in a given tissue relative to maximum expression and should therefore be less susceptible to low levels of noisy expression. It is tempting, therefore, to disregard the latter result. However, the adjusted coefficient of exchange recombination rate estimate (ACE; based on the local relationship between genetic and physical map positions) [35] gives the opposite result, with genes in clusters defined by both tau and expression breadth exhibiting elevated recombination rates (for tau median cluster rate = 1.2100, overall median rate = 1.1800, Wilcoxon test *P *= 0.0438; for breadth clusters median = 3.021, overall median = 2.934, Wilcoxon test *P *= 0.0099).

We therefore find no unambiguous evidence that clusters of putative housekeeping genes have low recombination rates. This may be due, in part, to the limited resolution of the presently available recombination maps and accords with our previous observation that broadly expressed genes (that is, genes with low tau) cannot be conclusively shown to have elevated or decreased recombination rates given said maps [28].

### Characterizing co-expression clusters

#### Small co-expression clusters are functionally coordinated

**Figure 1:**
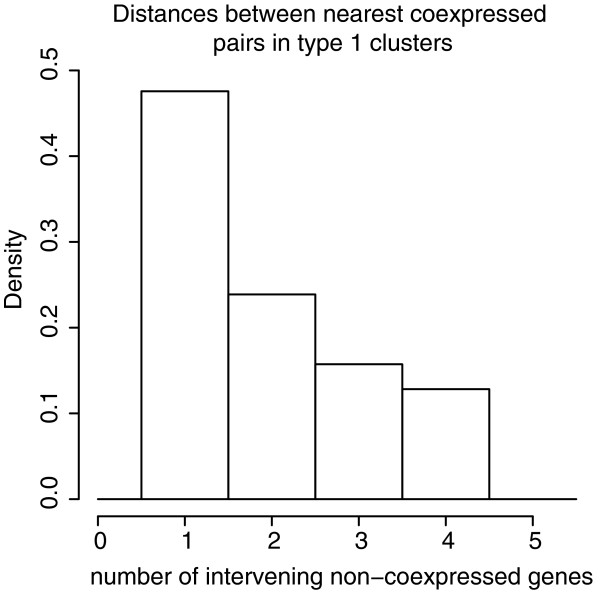
**Frequency distribution of the number of intervening non-coexpressed genes between the nearest co-expressed pairs within type 1 co-expression clusters**. Close to half of all co-expressed genes in clusters are immediately adjacent to their nearest co-expressed neighbors.

We next characterized gene clusters defined by high levels of co-expression using both a ten-gene sliding frame approach and a cluster-growing algorithm that does not impose a fixed window size. A compendium of all identified co-expression clusters and the genes therein is given in Additional file 3. The cluster-growing algorithm finds that most co-expressed genes are immediate neighbors or separated by just one other (non-co-expressed) gene (Figure 1) and clusters tend to be small in terms of both span and gene number (see Figure S1 in Additional file 4). As the null expectation is not skewed to detection of immediate neighbors, the distribution of distance between co-expressed genes provides *prima facie *evidence that such clusters may well be functionally important. As required by definition, the median co-expression in the type 1 clusters is very high (0.8644).

The relevance of SR clusters is not so immediately evident. As required by definition, co-expression between genes in SR clusters is, on average, higher than between random genes (median for SR clusters = 0.303; Wilcoxon test difference = -0.2275, *P *< 2.2 × 10^-16^). However, out of all possible pairwise expression correlations between genes in each SR cluster, a striking 30.06% have a negative Pearson's correlation coefficient. Naturally, this is lower than expected for gene pairs selected at random (10,000 per chromosomal class), where 48.01% show a negative correlation (median of 0.0295), but nonetheless indicates a large amount of negative co-expression in clusters that are putatively positive co-expression domains.

Previous evidence from various taxa supports the view that gene clusters that are functionally coordinated are also similar in terms of their expression patterns. For instance, in multiple species, including *D. melanogaster*, co-expression between clustered genes belonging to the same GO classification has been reported to be stronger than co-expression between functionally coordinated genes that are not proximate [16]. Meanwhile, proximate genes in *S. cerevisiae *tend to be close in the protein interaction network, and proximity in the network predicts co-expression [36]. However, not all functionally coordinated clusters observed in *D. melanogaster *are simultaneously co-expression clusters [37].

We therefore asked whether clusters of genes with correlated expression profiles tend to be enriched for genes with similar functions. Spellman and Rubin [29] previously reported that the number of GO terms associated with co-expression clusters defined by a sliding window approach is no higher than expected after accounting for tandem duplication. However, the more relevant question, perhaps, is whether the number of clusters showing significant GO term enrichment is greater than expected (with or without tandem duplicate control). To determine whether the two types of co-expression cluster are functionally coordinated, we asked whether the number of clusters significantly associated with at least one GO Slim term was higher than expected. We find that both observed counts exceed the expected number (dynamic growth method observed counts 542, *P *< 0.0001; SR sliding-window method observed counts 160, *P *< 0.0001) based on 10,000 bootstraps. Therefore, both types of cluster show significant functional enrichment.

There might, however, be a trivial explanation for this. Duplicated *D. melanogaster *genes that have remained adjacent over evolutionary time are enriched for developmental and transcriptional regulatory functions, as well as being co-expressed [33]. While in some taxa only a minority of duplicated genes are co-expressed [38,39], to rule out the possibility that our observations are explained by tandem duplication we removed duplicates found within the same cluster. In this set, the observed number of GO-term-enriched clusters still exceeds what is expected by chance for the dynamic growth algorithm (454, *P *< 0.0001) but not the sliding window algorithm (observed 115, *P *= 0.4720). This suggests that the large SR clusters are not functionally coordinated and any appearance to the contrary is owing to tandem duplicates. Equally, we find no evidence that the presence of duplicates, using the cutoff defined by Spellman and Rubin [29], accounts for functional coordination of genes in clusters defined by our algorithm.

It ought to be noted that the tandem duplicates in *D. melanogaster *that remain adjacent in *Anopheles gambiae *(and thus show elevated co-expression and functional similarity) form only a small subset of all tandem duplicates (13.55%) [33]. We would hence not expect all tandem duplicates to be both highly co-expressed and functionally related and our results therefore need not conflict with the findings of Quijano *et al*. [33]. Moreover, as with the functionally enriched type 1 clusters, functional neighborhoods described in multiple species, that is, local enrichments for GO terms defined by a sliding window algorithm, are also not explained by tandem duplication [16].

These results indicate that failure to find evidence that SR co-expression clusters show functional coordination may have been partly due to the way the question was asked and partly due to the presence of duplicates. It should also be noted that Spellman and Rubin [29] filtered duplicates and then redefined co-expression clusters. This means that the clusters they are comparing may no longer be in the same locations. Their test does not thus directly address the question as to whether duplicates are responsible for functional coordination within co-expression clusters defined when not allowing for duplicates. Spellman and Rubin also appear to have completely deleted duplicate genes rather than maintaining one from each pair.

#### Non-independence of large and small co-expression clusters

As we noted above, the method to define SR clusters may well be an inexact method to detect the small type 1 clusters. Moreover, Spellman and Rubin [29] suggest that the presence of large domains of coordinated expression provides evidence that chromatin status drives co-expression. However, this assumes that the size of the clusters defined by their algorithm is not merely a result of the method employed. We therefore compare cluster sizes of SR clusters with those of chromatin-defined units and ask whether large and small co-expression clusters are independent.

What, then, is the relationship between small and large clusters, that is, do small type 1 clusters tend to be subsets of their larger SR sliding window counterparts? The observed proportion of genes in small clusters that fall completely within large SR clusters is 0.3506. To determine whether this proportion was greater than expected, the locations of small clusters were held fixed while large clusters were randomly assigned a location in the genome. The maximum expected proportion of small-cluster genes falling into larger clusters was 0.2428 based on 10,000 randomizations. Therefore, the extent of overlap between small and large clusters is greater than expected by chance. In comparison, type 1 co-expression clusters do not overlap housekeeping clusters more often than expected by chance for either the tau definition (observed proportion of genes in co-expression clusters that fall entirely within a housekeeping cluster = 0.1068, *P *= 0.1292) or the breadth definition (observed proportion = 0.2444, *P *= 0.08).

As expected, if most SR clusters are picking up small clusters, 79.06% of SR clusters contain one or more small clusters. The majority of the large clusters (56.84%) contain just one small cluster (median number of overlapping type 1 clusters = 1). The number of overlapping small clusters increases with SR cluster size (Spearman's rho = 0.4418, *P *= 1.348 × 10^-12^, *n *= 234). Altering the cluster-growing algorithm to allow only co-expression clusters without gaps does not affect the finding that SR and type I clusters are not independent (see Supplementary Information R1 in Additional file 4). Given the extent to which SR clusters are overlapped by type 1 clusters, it is likely that the presence of a few strongly co-expressed genes leads to the sliding frame algorithm picking up greater-than-expected correlations across the whole window, and that large cluster size is indeed an artifact of the employed methodology. This idea is also supported by the overall lower (and indeed negative) co-expression we observed in SR clusters compared to type 1 clusters. Based on these results, one could argue that sliding frame clusters contain more 'junk', that is, fewer co-expressed genes, and are hence not an appropriate choice for investigating whether co-expressed proximate genes are functionally coordinated. While we have no doubt that SR clusters can be defined, we see no evidence to suppose that, as defined, they are biologically meaningful units. This accords with the observations of Sémon and Duret [14], who report that most mammalian co-expression clusters, defined as a contiguous group of co-expressed genes, contain only two genes.

What then of the claim that the size of SR clusters provides *prima facie *support for an involvement of chromatin [29]? We observe a median cluster size of 13 for the approach of Spellman and Rubin [29]. The chromatin domains reported by de Wit *et al*. [37] contained a median of 26 genes, that is, twice as many. However, median cluster size is 3 for type 1 clusters (Figure S1 in Additional file 4). Although our clustering algorithm allows gaps of up to four genes within clusters, 47.57% of cluster members are adjacent to another cluster member with no intervening non-co-expressed genes (Figure 1). For the duplicates-removed sets, median cluster sizes are 11 and 3, respectively. This questions the assertion that cluster size is, by itself, indicative of large open chromatin regions permitting gene expression.

However, for both types of co-expression cluster we can find evidence that chromatin level effects may well be relevant. If co-expression clusters are correlated in terms of their expression patterns due to co-regulation as opposed to chromatin effects, we might expect to see no decrease in co-expression scores with increasing distance between non-overlapping genes belonging to the same cluster. If co-expression is owing to local relaxation of chromatin, we might expect to see weaker co-expression with greater distance. For both SR and type 1 clusters, we observe a negative correlation (assayed by Spearman's rho, hereafter referred to as rho) between IGD and co-expression (rho = -0.1176, *P *< 2.2 × 10^-16^, *n *= 31,127 for SR clusters; rho = -0.0596, *P *= 1.102 × 10^-5^, *n *= 5,438 for type 1 clusters), as well as distance between midpoints and co-expression (rho = -0.1287, *P *< 2.2 × 10^-16 ^for SR clusters; rho = -0.0733, *P *= 6.236 × 10^-8 ^for type 1 clusters). That co-expression between genes in clusters decays with distance is consistent with chromatin effects facilitating co-expression of genes that are close together and accords with previous reports of a relationship between intergenic distance and co-expression levels [20,40-43].

We can further examine the possibility that chromatin effects are responsible, in part, for co-expression of adjacent genes by comparing our clusters with clusters that are more likely to show co-regulation. Mezey *et al*. [1] provide a list of sliding-window-defined clusters of genes that are physically adjacent in *D. melanogaster *and whose mean expression levels are correlated between males of seven species of the *D. melanogaster *subgroup, that is, genes showing evolution of coordinated expression. We therefore ask to what extent small co-expression clusters share genes with between-species clusters defined by Mezey *et al*. [1] using a ten-gene window, removing any genes withdrawn from FlyBase (FB 2010_03). A small percentage (7%) of small co-expression clusters share one or more genes with the between-species clusters, with 5% of genes in small co-expression clusters also being found in a between-species cluster. This accords with the finding that co-expression clusters defined within *Drosophila simulans *do not correspond to those defined between species [1] and argues against co-expression of adjacent genes in a given species being due to co-regulation. By contrast, around half (51%) of all tau clusters share at least one gene with between-species clusters, with 27% of genes in tau clusters also being present in a between-species cluster (but only 9% of between-species cluster genes also being present in a tau cluster). This result is, again, consistent with small co-expression and housekeeping clusters comprising different sets of genes that are regulated differently, with a subset of putative housekeeping genes possibly showing coordinated evolution across multiple *Drosophila *species.

#### Divergent gene pairs may be unusually common in small co-expression clusters

Transcriptional orientation is thought to influence co-expression of gene pairs. Conserved divergently paired housekeeping genes have been reported in *D. melanogaster *[44] and it has been proposed that such pairs show increased co-expression [20,45] as well as functional coordination, with bidirectional pairs with similar functions showing more strongly correlated expression [46,47]. We therefore ask whether divergently oriented gene pairs are overrepresented in adjacent gene pairs in co-expression clusters. Chi squared is borderline significant for small type 1 clusters (32% divergent clusters) versus all genes (29% divergent clusters; chi squared 7.7374, df = 3, *P *= 0.0518) but not sliding window SR clusters (28% divergent clusters) versus all genes (chi squared 5.6318, df = 3, *P *= 0.1310). These numbers are roughly in accord with the reported 31% divergently oriented genes in *D. melanogaster *gene pairs with intergenic distance <600 bp [48]. Based on the small difference in percentages and the marginally significant test, there is some evidence that divergently oriented pairs are more common within small co-expression clusters. This is consistent with the possibility that co-expression is partly owing to divergent transcription, as has been observed in humans [49].

### Gene order evolution

Before we can assess whether gene clusters show greater than expected evolutionary conservation due to selection, we must identify potential covariates. Compared to mammals [19], the genus *Drosophila *shows a high degree of genome rearrangement [50]. Functional constraint is thought to be responsible for only around 15% of gene order conservation [19], as reuse of linkage breakpoints is influenced by the fragility of certain types of sequence (see, for example, [51,52]). We start by examining the role of recombination and IGD in modulating rearrangement rates.

#### Recombination is associated with gene order rearrangement

One force affecting gene order conservation is recombination. Recombination is associated with a higher rate of chromosome rearrangement in wheat [30] and zebra finch, suggesting a possible role of non-allelic homologous recombination (also termed ectopic recombination) in structural rearrangement [31]. Consistent with this idea, adjacent *S. cerevisiae *gene pairs specifying proteins with network proximity only remain linked in *Kluyveromyces lactis *when recombination between the loci is unusually low [36].

**Figure 2:**
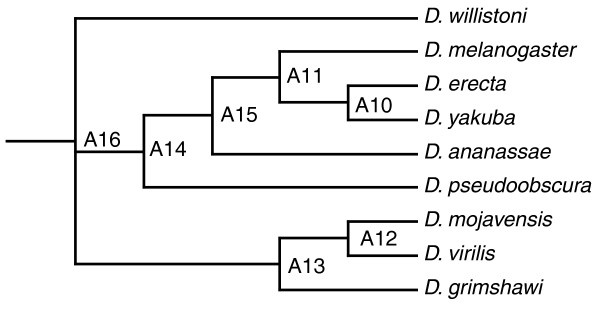
Reconstructed ancestral nodes A16 and A13 were used to define *D. melanogaster *gene pairs with ancient linkage that were rearranged on the branch leading up to *D. mojavensis*, *D. virilis and D. grimshawi*

We therefore ask whether recombination is also associated with an increase in linkage breakage in *D. melanogaster*. Von Grotthuss *et al*. [19] provide information on blocks of genes conserved between nine *Drosophila *species (Figure 2). Linkage blocks were defined using the gene order and orientation synteny definition, which requires both gene order and orientation to be maintained across the species tree [19]. The correlation between linkage block size and recombination [53] is not significant (rho = -0.0268, *P *= 0.1364 for RP; rho = -0.0276, *P *= 0.1262 for ACE; *n *= 3,080). However, when non-recombining blocks (defined as having a mean RP rate of 0) are excluded a significant negative relationship between mean recombination rate and block size is observed (rho = -0.0411, *P *= 0.0301 for RP; rho = -0.0419, *P *= 0.0268 for ACE; *n *= 2,788). Hence, we conclude that conserved blocks in regions of low recombination, at least as defined in *D. melanogaster*, tend to be longer than blocks in regions of high recombination despite the low resolution of available measures of recombination. Additionally, recombination rates positively correlate with the average breakpoint index (rho = 0.0427, *P *= 0.0179 for RP; rho = 0.1017, *P *= 1.543 × 10^-8 ^for ACE; *n *= 3,080), that is, the number of breaks at either edge of each conserved block divided by two, indicating that the process of recombination might increase the rate of rearrangement.

A decrease in block length is observed in regions of high recombination regardless of how conserved blocks are defined. When we consider only rearrangements that have occurred between the inferred ancestor of all nine species and *D. melanogaster*, or only rearrangements in the lineage leading up to *Drosophila grimshawi*, *Drosophila mojavensis *and *Drosophila virilis *for blocks conserved between the ancestor and *D. melanogaster*, similar results are obtained (Supplementary Information R2 in Additional file 4). We thus conclude that gene order rearrangement is associated with elevated recombination rates.

#### Short intergene distance predicts a high rate of gene order rearrangement

Given prior observations from yeast [11], the size of the space in between pairs might also be expected to influence the degree of conservation, as longer intergene space may provide more opportunity for rearrangement without disrupting function. Does IGD predict gene order rearrangement between neighbors?

For each pair of adjacent genes with available linkage information, IGD was calculated (including UTRs). Overlapping gene pairs and pairs whose intergenic region was overlapped by another gene were filtered out, as rearrangements between these pairs would involve the disruption of coding region or UTRs; 4,912 pairs remained. We then encoded whether or not both genes in a pair reside in the same conserved block across the species tree (Figure 2) using the gene order and orientation synteny definition that requires both gene order and orientation to be maintained. If IGD affects the degree of conservation by providing opportunities for rearrangement, we might expect to see a greater proportion of pairs with small IGD with conserved linkage.

**Figure 3:**
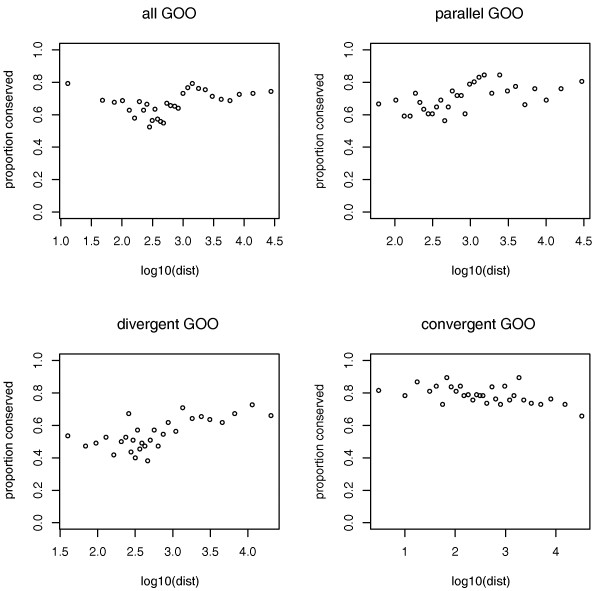
**The proportion of gene pairs with conserved linkage, as defined by residing in the same orthologous landmark, increases with increasing IGD, except for convergently oriented gene pairs, where greater IGD predicts a lower rate of conservation consistent with more opportunities for rearrangement**. GOO, gene order and orientation synteny definition.

Gene pairs were separated into 30 bins of approximately equal size according to IGD and the proportion of matched blocks, that is, instances of linkage conservation across the species tree, was determined for each bin. However, the positive slope obtained for distance versus proportion conserved is not monotone (Figure 3). Pairs were thus further separated into divergent (*n *= 1,656), convergent (*n *= 1,124) and parallel orientations (*n *= 2,132), as orientation might also be expected to influence the degree of linkage conservation. A negative monotone slope was obtained for convergently transcribed pairs. The negative correlation (rho -0.5413, *P *= 0.0020) indicates that short IGD predicts a higher degree of linkage conservation in this subset, consistent with the idea that longer IGDs provide more opportunity for rearrangement without disrupting function. However, contrary to our expectation, the remaining orientations appear to show a non-monotone positive relationship between IGD and linkage conservation (rho = 0.4316, *P *= 0.0173 for all orientations; rho = 0.6309, *P *= 0.0002 for parallel; rho = 0.6227, *P *= 0.0002 for divergent; Figure 3). Note, however, that Spearman's rho is not suitable for detecting non-monotone relationships.

#### Co-expression predicts high rearrangement rates

How might we account for the counterintuitive observation that gene pairs with shorter IGDs (for all orientations but convergent) are rearranged at a higher rate despite having less 'opportunity' to do so? One possibility is that these pairs are more highly co-expressed by accident of being in the same open chromatin at the same time, which has been argued to have a deleterious effect [15]. This is indeed what we observe in *D. melanogaster*. Intergene distance negatively correlates with co-expression (rho = -0.0473, *P *= 0.0009, *n *= 4,912), but only for divergently transcribed pairs (rho = -0.1634, *P *= 2.237 × 10^-11^) and not convergent (rho = -0.0045, *P *= 0.8807) or parallel pairs (rho = 0.0012, *P *= 0.9544). This is presumably owing to shared *cis*-regulatory elements located in the intergenic regions of divergently transcribed genes. Median co-expression is lower for convergently transcribed pairs (median = 0.1180) than for parallel (median = 0.1683) and divergent pairs (median = 0.2945). That divergent pairs have higher co-expression has also been observed in mouse and human [40], and is consistent with the idea that bipromoters facilitate co-expression of linked genes [49]. Additionally, reduced co-expression of convergently oriented genes has been reported in zebrafish [42]. Meanwhile, linkage conservation shows the opposite pattern: convergent pairs are 78.83% conserved (238 of 1,124 rearranged), compared to 70.59% (627 of 2,132 rearranged) for parallel and 54.89% (747 of 1,656 rearranged) for divergent pairs. Divergent pairs are significantly overrepresented in the rearranged set (chi sq = 193.8161, df = 2, *P *< 2.2 × 10^-16^). These results are the opposite to what is seen in yeast [17].

As closely linked pairs appear to be maintained at a lower rate than more distant pairs, we then examined the effect of high co-expression on maintenance of linkage. Given their IGD, are the 5% most highly expressed pairs more or less likely to be rearranged? We employed a logistic regression model with conserved linkage as the outcome and IGD and high co-expression (defined as being in the top 5%) as the predictors. The best fitting model includes both predictors, as residual deviance is 6202.7 for a model with both predictors, 6211.1 for co-expression alone and 6208.7 for IGD alone, with a null deviance of 6217.4. Neither IGD (chi-square *P *= 0.0038) nor co-expression (chi-square *P *= 0.0149) can be removed to improve the fit. IGD predicts an increase in the proportion of matches (estimate 1.264 × 10^-5^, *P *= 0.00742) and co-expression a decrease (estimate -3.296 × 10^-1^, *P *= 0.01378). Hence, we find no evidence that greater IGD provides more opportunity for rearrangement, nor that highly co-expressed pairs are maintained by selection; rather we see the opposite trend.

The overall lower co-expression levels and lack of correlation between IGD and co-expression might explain why convergently transcribed pairs are unique in showing a decrease in gene order conservation with increasing IGD, consistent with the idea that longer IGDs provide more opportunities for rearrangement. Indeed, when only non-convergently oriented genes are considered in the logistic model, high co-expression no longer predicts conservation independent of IGD (estimate = -1.468 × 10^-1^, *P *= 0.3208 for co-expression; estimate = 2.239 × 10^-5^, *P *= 0.0002 for IGD; *n *= 3,788). On the other hand, when only convergent pairs are considered in the logistic model, co-expression remains the only significant predictor of linkage conservation (estimate = -1.048, *P *= 0.0010 for co-expression; estimate = -1.091 × 10^-5^, *P *= 0.1775 for IGD; *n *= 1,124).

Thus, it appears that genes with short IGD tend to be more highly co-expressed, depending on orientation, and more likely to be rearranged, with the most highly co-expressed pairs showing increased rates of rearrangement, with the exception of convergently oriented pairs where high co-expression alone predicts reduced linkage conservation.

The above results contrast strikingly with prior results in yeast [11] as well as with some prior observations in mammals [13,14] and plants, where short IGD is a major predictor of linkage conservation [54]. However, a more recent study reported, as we observe, a reduced rate of linkage conservation in linked co-expressed gene pairs and attributed this to potentially disadvantageous effects of leaky expression [15]. The discrepancy between this result and those of previous reports may be explained by the lack of an outgroup in the earlier studies. Without polarizing gene order rearrangements it is impossible to distinguish between the formation of new and the destruction of old linkages [15]. Indeed, when Singer *et al*.'s [13] data are reanalyzed using an outgroup, the data no longer support the notion that co-expressed clusters are maintained by selection because they are advantageous [15].

To address whether consideration of the polarity of changes might alter our results, we asked whether closely linked - and thus presumably more highly co-expressed - gene pairs in *D. melanogaster *are more likely to have undergone gene order rearrangement in a particular lineage. We find that considering whether pairs adjacent in *D. melanogaster *were either newly linked, that is, not adjacent in the ancestral state, or conserved between the ancestral state and *D. melanogaster *but broken up in the lineage leading up to *D. grimshawi*, *D. mojavensis *and *D. virilis *does not affect our conclusion (see Supplementary Information R3 in Additional file 4; Figure 2).

#### Co-expression is not directly associated with recombination

It is tempting to speculate that selection against (potentially leaky) co-expression is responsible for the increased rate of rearrangement. However, it does not necessarily follow that closely linked or highly co-expressed pairs are rearranged because they are disadvantageous. Given that we can establish that recombination is associated with gene order rearrangement and co-expressed pairs tend to be more highly rearranged, we need to rule out the possibility that high recombination between co-expressed genes is responsible for the observed synteny breaks. Indeed, recombination rates are known to be elevated for highly co-expressed linked genes in humans [15].

We find no evidence for an association between co-expression and mean recombination rates of adjacent gene pairs in *D. melanogaster *(rho = -0.0113, *P *= 0.4317 for ACE; rho = 0.0058, *P *= 0.6838 for RP; *n *= 4,879). Although failure to detect such an association could be partly due to the lack of high-resolution recombination data, we conclude that there is presently no evidence that increased recombination is responsible for reduced maintenance of co-expression clusters in *D. melanogaster *or disruption of adaptive gene order [36]*.*

### SR clusters and clusters of broadly expressed genes are not more likely to be conserved than expected

In light of the absence of evidence for an association between housekeeping clusters and low recombination rates, we might expect to see no unusual patterns of gene order conservation in housekeeping clusters. We therefore examine whether these clusters are more likely than expected to show an increase in gene order conservation than expected. We compared each cluster to 1,000 randomly selected stretches with the same number of genes that did not overlap with a cluster by counting the number of orthologous landmarks present in each cluster as a proxy for rearrangements. We observed no increase in gene order conservation for either clusters defined by tau (*P *= 0.2337) or number of tissues expressing a given gene (*P *= 0.2503). This was also the case when we counted only rearrangements in A13 (that is, the node leading up to *D. grimshawi*, *D. mojavensis *and *D. virilis*; Figure 2) as breaks in gene order (*P *= 0.3842 for tau clusters; *P *= 0.4535 for breadth clusters). We therefore find no evidence that selection acts to preserve clusters of putative housekeeping genes.

Given the non-independence between SR and type 1 clusters, the weak median co-expression levels in SR clusters compared to type 1 clusters and the extent to which negative co-expression scores between genes in SR clusters are detected, we concluded above that SR clusters are likely to be artifacts of the underlying method rather than true co-expression clusters. Not only are a substantial proportion of genes assigned to these clusters presumably irrelevant to the putative cluster, but given the lack of conservation between highly co-expressed adjacent genes described above, we might not predict SR clusters to show reduced rates of linkage breakage even if they were true co-expression clusters. This is indeed what we observe when we repeat the above test. SR clusters are not less rearranged than randomly selected stretches of genes of equivalent size (*P *= 0.52 for orthologous landmark numbers; *P *= 0.4749 for breaks in A13 order). We therefore find no evidence in *D. melanogaster *that genes in clusters defined by similarity in expression patterns are conserved as units of evolution.

## Discussion

In terms of the types of clusters observed, *D. melanogaster *appears to resemble the model organism for gene cluster analysis, *S. cerevisiae*. We find that, like yeast, *D. melanogaster *contains small co-expression clusters that show evidence of functional similarity. *D. melanogaster *also has type 2 (housekeeping/essential) clusters, although it is not clear that such housekeeping clusters in *D. melanogaster *have reduced recombination rates. The previously described larger SR co-expression clusters, not yet described in yeast, are likely an artifact of imposing a fixed window size and the presence of the type 1 clusters that fall entirely within SR clusters.

This is largely where the similarities end. Despite the fact that domains of high recombination tend to be more highly rearranged in both species [36], there are also striking differences between yeast and *D. melanogaster *in terms of gene order conservation. Yeast shows a reduction in gene order rearrangements between genes in housekeeping and co-expression clusters [10,11]. Meanwhile, we find no evidence that either type of cluster is maintained at a higher rate than expected in *D. melanogaster*. On the contrary, we observe that highly co-expressed neighboring genes are slightly less likely to be conserved than other adjacent genes. This contrasts with previous reports of clusters defined by chromatin status in *D. melanogaster*, such as those described by de Wit *et al*. [37], which are enriched for co-expression and exhibit a dearth of synteny breaks, as well as functionally coordinated clusters of genes that are regulated by specific chromatin regulators and conserved in other insect species [55]. Although Bhutkar *et al*. [56] report that around 60% of genes belonging to the same synteny block (calculated across nine *Drosophila *species) show positively correlated embryonic expression, their analysis did not account for the relationship between physical proximity and co-expression documented above. On the other hand, clusters of genes that are physically adjacent in *D. melanogaster *and whose expression patterns correlate highly between males of seven *Drosophila *species, and which might therefore be expected to be co-regulated [1], are no more conserved than expected. Indeed, they are broken in more than half of all cases [19]. Additionally, we observe a general trend for divergently paired and more closely linked genes to become rearranged.

Our findings seem consistent with the notion that much co-expression of linked genes is disadvantageous and likely due to transcriptional interference [15]. The observation that similarity in expression patterns within type 1 and SR clusters tends to decay with increasing distance between genes is also consistent with co-expression being, in part, a consequence of shared chromatin as opposed to tight co-regulation. That housekeeping clusters cannot be shown to have either increased gene order conservation or reduced recombination rates could also point to the 'leaky expression' model. It is, however, likely that the limited resolution of currently available recombination maps affected our ability to detect reduced levels of recombination [28].

How can the unexpected finding that pairs of neighboring genes in co-expression clusters are rearranged slightly more often than adjacent pairs not found in clusters be reconciled with the finding that genes in co-expression clusters show functional similarities not explained by tandem duplication? Recent evidence from *D. melanogaster *indicates that gene order within clusters of testis-expressed genes or nuclear proximity need not be maintained to ensure co-expression, although it could facilitate more efficient co-expression [57]. It has also recently been suggested that an increased rate of genome rearrangement does not necessarily indicate selection to break apart gene pairs. Rather than being conserved in terms of their gene order, gene neighborhoods with homologous functions shared between humans and chimpanzees are enriched for synteny breaks [16]. While the neighborhoods themselves are conserved between species, the genes found within them are not necessarily orthologous. This would appear to suggest that selection acts to preserve functional neighborhoods rather than ancestral gene composition and order, and a gene order break in a given neighborhood would be followed by new rearrangements to restore the functional unit [16]. This model can be reconciled with our observation that functionally coordinated co-expression clusters show increased levels of gene order rearrangement and that pairs with short IGD are more likely to have originated recently (Supplementary Information R3 in Additional file 4). Possible support for the notion that co-expressed pairs with common functions are generally prone to gene order disruption comes from *S. cerevisiae*. It is suggested that proximity facilitates co-regulation by bi-directional promoters or common transcription factors, but also favors an increase in the recombination rate, eventually leading to gene order rearrangement [36].

The question remains why co-expression is not an independent predictor in the logistic regression models for recently formed pairs and pairs broken in A13 (Supplementary Information R3 in Additional file 4), unlike when breaks across the whole species tree are considered. This might be due to a lack of depth, that is, an insufficient number of synteny breaks between closely related species when only certain classes of rearrangement are considered. Moreover, a lack of data in species other than *D. melanogaster *limits our ability to assess whether co-expression levels have changed after rearrangements in the branch leading up to A13 (Supplementary Information R3 in Additional file 4), as well as our ability to look for co-expression clusters that show functional homology with those found in *D. melanogaster*. Expression pattern divergence for sex biased drosophilid genes is known to increase with divergence time, with different genes contributing to this effect to differing degrees [58]. We might therefore also expect the expression pattern of a given gene, and thus the degree to which it correlates with expression patterns of neighboring genes, to have changed in species other than *D. melanogaster*, thus adding noise to our gene order rearrangement analysis.

Additional limitations may arise from the limited availability of recombination rates for species other than *D. melanogaster*. The extent of recombination rate divergence between species depends on scale, with fine-scale rates tending to diverge more rapidly than broad-scale rates (see, for example, [59-61]). Although this could introduce some noise to our analysis of the possible effects of recombination on gene order rearrangement, the resolution of the rates available for *D. melanogaster*, particularly RP, is limited in terms of reflecting fine-scale variation. Additionally, there is evidence that fine-scale recombination rates between the drosophilid sister species *D. pseudoobscura *and *D. persimilis *are highly correlated [62].

## Conclusions

The genome of *D. melanogaster *bears some resemblance to that of *S. cerevisiae*, the model species for studying gene order, in terms of the presence of both co-expression and housekeeping/essential clusters that have similar dimensions and patterns of functional similarity. Nevertheless, the striking differences in patterns of gene order conservation that we observe strongly caution against the assumption that what applies for one well-characterized model organism may be generalized to other species or taxa. While in yeast the clusters are conserved as syntenic units more than expected by chance, if anything in *D. melanogaster *the opposite is seen. The picture in *D. melanogaster *seems consistent with co-expression being due, in part, to shared chromatin environment and indeed potentially disadvantageous.

## Materials and methods

### SR co-expression clusters defined by fixed-size ten-gene sliding window approach

We defined gene clusters using a sliding window approach as in Spellman and Rubin [29]. Using Pearson's correlation coefficient, mean co-expression for all possible pairwise combinations was computed for each window of ten adjacent genes. For each chromosomal class (4, X, 2L, 2R, 3R, 3L), the mean Pearson's correlation coefficient for all possible pairwise combinations for every ten-gene window was calculated. A threshold value for each chromosomal class was defined by calculating the means from 10,000 sets of 10 randomly selected genes as above, and setting the 97.5th percentile as the cutoff. Where the correlation for a window exceeded the threshold value, the genes contained within that window were defined as belonging to a cluster. Adjacent or overlapping clusters were merged, as done by Spellman and Rubin [29].

#### Dynamic co-expression clustering algorithm

To overcome the limitations posed by the above approach, we developed an algorithm that does not rely on a fixed window size to assign genes to co-expression clusters, as well as allowing gaps of up to three genes within those clusters.

Pearson's correlation coefficient was used to determine co-expression of genes from the Spellman and Rubin [29] dataset. In order to determine a cutoff, 100,000 random gene pairs from each chromosomal class (4, X, 2L, 2R, 3R, 3L) were correlated. For genes as or more strongly correlated as the top 5% of randomized values (note that we use a stricter threshold for SR clusters), co-expression was considered higher than expected by chance. The beginning of a cluster is defined as a pair of genes that meets or exceeds the threshold and is no farther than three genes apart. The cluster is expanded where genes no farther away than the permitted gap size were found to have an average correlation with all existing cluster members (not including those genes not co-expressed with others) that met or exceeded the threshold, or showed an above-threshold correlation with the last gene added to the cluster. Hence, as opposed to Spellman and Rubin's [29] algorithm, all genes that are added to a cluster are required to correlate with genes that are already cluster members. Those genes that do not meet these criteria are not considered part of the cluster, even if they are physically within the limits of a cluster.

#### GO Slim enrichment analysis

As associations are more likely to be detected for broader categories than terms associated with a small set of genes, to determine whether co-expression clusters are functionally related, GO terms were mapped to GO Slim using map2slim.pl (part of the go-perl package). To detect clusters significantly enriched for GO Slim terms, the cumulative hypergeometric distribution was employed:(1)

where *P *represents the probability of finding *k *or more genes associated with a particular GO Slim term in a cluster of size *n *(not including uncorrelated genes), out of a total of *A *genes associated with that term on the same chromosomal arm, from a total of *G *genes present on the chromosomal arm [63]. To determine whether the number of clusters significantly enriched for at least one term exceeded random expectation, all genes in clusters were replaced with randomly selected genes from the same chromosomal class (10,000 replicates).

#### Removal of duplicated genes

Duplicates within gene clusters were defined by comparing protein sequences using Blastp with an e-value of <10^-7 ^(as in Spellman and Rubin's analysis) filtering low complexity regions [64]. For each duplicate pair, one gene was randomly deleted. Clusters with only one remaining gene after removal of duplicates were deleted.

#### Expression breadth

Tau, a measure of tissue specificity that takes account of how many tissues a given gene is expressed in, as well as the expression level relative to maximal gene expression [65,66], was calculated using adult tissue expression data from Fly Atlas [32]:(2)

*logS(j) *for a given tissue was set to 0 where expression was only detected on one array for that tissue. Genes where all tissues were set to 0 were removed from the analysis. The influence of large differences between maximal gene expression and tissue expression is reduced by logging the ratio of tissue expression to maximal gene expression.

#### Recombination rates

Per-gene estimates of recombination derived from release 4.3 of the *D. melanogaster *genome were obtained from Larracuente *et al*. [53]. Here we use recombination rates calculated by two different methods: ACE is based on the relationship between genetic and physical map positions across polytene bands, that is, on a local scale; RP is calculated from the slope of the third order regression polynomial at the midpoint of each gene [35,67].

#### Statistics

All statistical analyses were performed in R.

## Abbreviations

ACE: adjusted coefficient of exchange recombination rate estimate; GO: Gene Ontology; IGD: intergene distance; RP: regression polynomial recombination rate estimate; SR: Spellman and Rubin; UTR: untranslated region.

## Competing interests

The authors declare that they have no competing interests.

## Authors' contributions

CCW compiled, processed and analyzed the data. LDH conceived the study. CCW and LDH wrote the manuscript. All authors read and approved the final manuscript.

## Supplementary Material

Additional file 1 FlyBase identifiers for breadth clusters. Click here for file

Additional file 2 FlyBase identifiers for tau clusters. Click here for file

Additional file 3 FlyBase identifiers for small co-expression clusters. Click here for file

Additional file 4 Supplementary results and Figures. Click here for file
