# Supplementary material for: Support for multiple classes of local expression clusters in Drosophila melanogaster, but no evidence for gene order conservation
Source: Genome Biol. 2011 Mar 17;12(3):R23. doi: 10.1186/gb-2011-12-3-r23 (PMC3129673; doi:10.1186/gb-2011-12-3-r23)
Supplement: Additional file 4 — Supplementary results and Figures. [file gb-2011-12-3-r23-S4.PDF]

Supplementary information

Results

R1. Disallowing gaps in coexpression clusters does not affect the conclusion that SR clusters are not independent of type I clusters

When no gaps in type I coexpression clusters are permitted, median cluster size decreases from 3 to 2. As before, the proportion of genes in type I clusters that fall entirely within an SR cluster is greater than expected by chance (observed proportion 0.4951, maximum randomised value = 0.3735, $p < 10^{-4}$ for 10000 iterations). 77.35% of SR clusters are overlapped by at least one type I cluster, with 34.62% of SR clusters containing a single type I cluster and 29.91% containing two. Therefore, removing gaps does not affect the conclusion that SR clusters are presumably a method artefact.

R2. Recombination is associated with gene order rearrangement regardless of the definition of linkage breakage employed

Does the negative relationship we observed between linkage block length and recombination rate also hold when conservation is defined based on rearrangements between the inferred ancestral order and *D. melanogaster*? We collapsed orthologous landmarks that are in the same order in A16, the reconstructed ancestral node of the species tree according to the GOO definition (see Figure 2; column A16 in Supplemental Data 1 [19]), and *D. melanogaster*, averaged recombination rates across these collapsed blocks, and repeated the analysis. Recombination negatively correlates with block length for ACE (rho = -0.1030, p = 0.0071, n = 682) and is on the edge of significance for RP (rho = -0.0681, p = 0.0755). Note that there is a substantial sample size reduction after collapsing blocks and we would hence expect these results to be

somewhat weaker. This indicates that longer blocks with ancient linkage tend to have lower than average recombination rates.

We then repeated this analysis for blocks that are conserved between A16 and *D. melanogaster* but not A13 (i.e. the reconstructed ancestral node for *D. grimshawi*, *D. mojavensis* and *D. virilis*, see Figure 2; Supplemental Data 1 [19]), i.e. ancestrally linked blocks not preserved in A13. As before, conserved blocks containing fewer genes tended to have higher recombination rates (rho -0.0977, p = 0.0006, n = 1217 for ACE; rho = -0.0552, p = 0.0541 for RP; after removing non-recombining regions: rho = -0.1167631, p = 9.654×10^{-5}, n = 1110 for ACE; rho = -0.0651, p = 0.0302 for RP). Therefore, longer blocks with ancient linkage in *D. melanogaster* that have also been conserved in the node leading to *D. grimshawi*, *D. mojavensis* and *D. virilis* have lower than average recombination rates.

That the RP recombination rates show a slightly weaker relationship with gene order rearrangement than the ACE rates is presumably owing to the methods by which the rates themselves were calculated. ACE is based on local estimates of the relationship between genetic and physical map positions across polytene bands. Meanwhile RP is the slope of the third order regression polynomial at the midpoint of each gene (see [35]). The polynomial approach has a smoothing effect that may obscure regional variation and tends to overestimate telomeric recombination rates [67]. Thus, if recombination affects gene order rearrangements on a local scale, we would expect this to be less apparent for RP.

R3. Consideration of ancestral and current gene order does not alter the conclusion that gene pairs with short IGD tend to be rearranged more frequently

In order to identify gene pairs with "old" linkage, we compared present gene order in *D. melanogaster* to the reconstructed ancestral node of the species tree (see Figure 2; column A16 in Supplemental Data 1 [19]) and kept only those pairs that are adjacent (with no intervening genes [using the GOO definition]) in both. We then identified the subset of pairs that are adjacent in A16 and *D. melanogaster* but not node A13 of the species tree (i.e. the reconstructed ancestral node for *D. grimshawi*, *D. mojavensis* and *D. virilis*, see Figure 2) and defined them as non-conserved.

In accord with the above association between IGD and conservation across the species tree, we observe a positive correlation between conservation in A13 and IGD (rho = 0.5056, p = 0.0044), indicating that closely linked genes are more likely to have been rearranged in A13. As expected, logistic regression also indicates that ancestrally conserved pairs that are closely linked in *D. melanogaster* are less likely to be conserved in A13 (Estimate 3.175×10^{-5}, p = 0.0242, null deviance 2082.2, residual deviance 2075.0). These results accord with Liao and Zhang [15]. However, as we only have expression profile information for *D. melanogaster* we cannot directly assess the effect of rearrangement on potential reductions in coexpression and coexpression itself is not a significant predictor of conservation. Note too that IGD is as defined in *D. melanogaster* and we have no information on distance at the precise points before and after rearrangement occurred.

Moreover, when we consider the orientations of the pairs that are rearranged in A13, we observe a bias towards divergently paired genes being rearranged (137/1504 pairs i.e. 9.11% rearranged) compared to convergent (46/1087 i.e. 4.23% pairs rearranged) and parallel pairs (92/2006, i.e. 4.59% rearranged; chi sq 39.0169, df = 2, p = 3.37 x

10^{-9}). This is consistent with the overall lower conservation of divergent gene pairs across the whole tree.

Pairs that are linked in *D. melanogaster* but not A16 indicate the formation of new linkages. We recorded the number of instances within each cluster where orthologous landmarks that are adjacent in *D. melanogaster* are not adjacent in the ancestral node A16 and defined these as "new", i.e. recently rearranged, pairs. The proportion of newly formed pairs is relatively low at (315/4912 pairs with 1612 pairs belonging to different orthologous landmarks; consistent with a total of 683/3101 blocks being discontinuous in the ancestor compared to *D. melanogaster*).

The relationship between transcriptional orientation of gene pairs and gene order rearrangement is also observed for newly formed pairs. Here, conservation indicates the persistence of old linkages as opposed to the formation of new linkages. Convergent pairs are 96.71% (37/1124 rearranged) conserved, compared to 94.09% (126/2132 rearranged) for parallel and 90.82% (126/1656 rearranged) for divergent pairs. Proportions differ significantly between conserved pairs and rearranged (chi sq = 40.2502, df = 2, $p = 1.819 \times 10^{-9}$) and divergent pairs are found among the new pairs more often than expected.

Are newly formed pairs unusual with respect to IGD? After assigning the data to 30 equal-sized bins according to IGD, conservation and IGD do not correlate in this set (rho = 0.1140, p = 0.5485). However, when we repeat the logistic regression we observe the same result as above: The proportion of pairs that have remained adjacent as opposed to being newly linked compared to the reconstructed ancestral order is

increased with greater IGD (Null deviance: 2339.9; Residual deviance: 2332.8; estimate = 2.911×10^{-5}, p = 0.0242; coexpression can be dropped from the model p chisq = 0.1586).

It is difficult to imagine why newly formed pairs should be arranged particularly close together and in divergent orientation. This might, therefore, suggest that such pairs tend to be newer because they are less likely to be maintained in this configuration for extended time spans. We conclude that gene pairs that are closer together are more likely to have been rearranged in the past. In addition, there is a general trend for divergently oriented genes to have been rearranged irrespective of the definition of rearrangement employed.

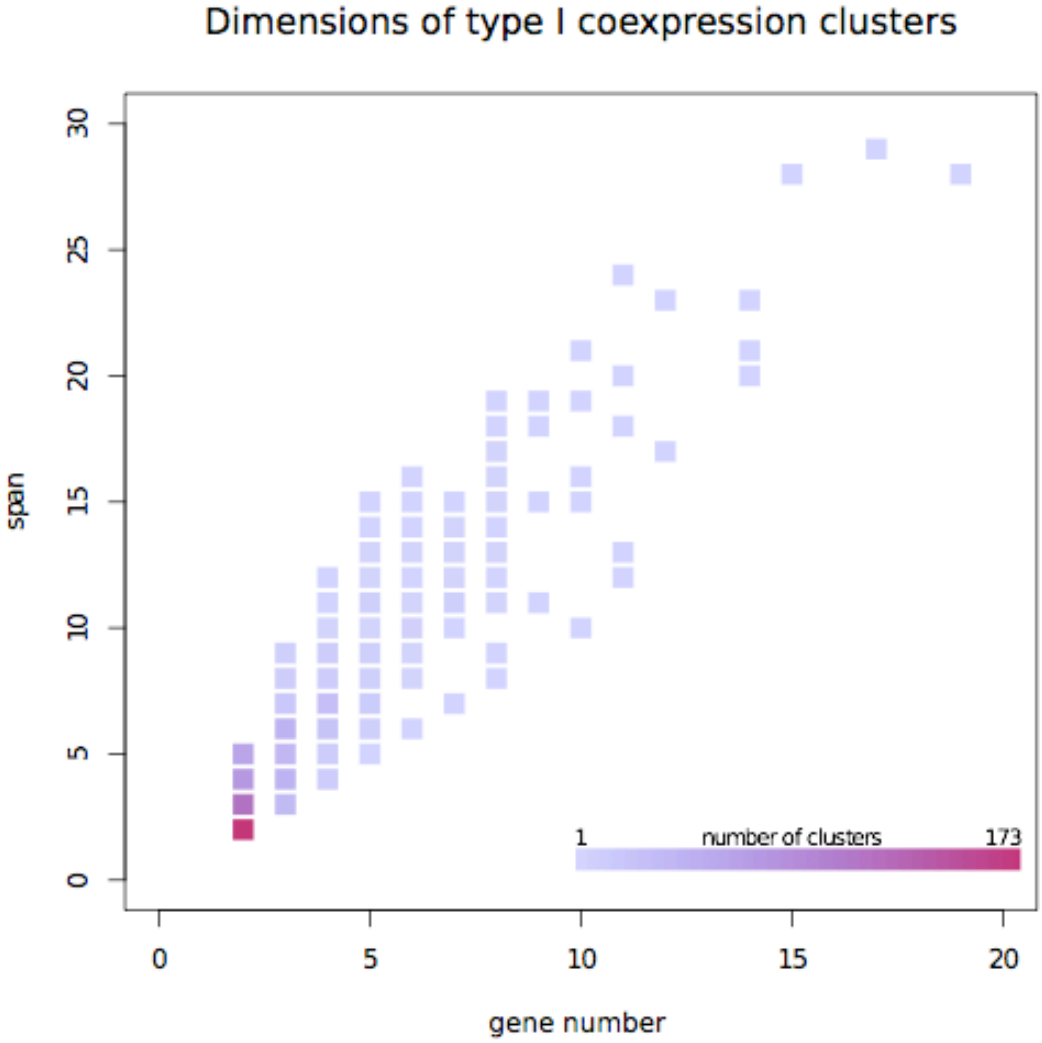

Supplementary Figure 1

Despite the fact that our algorithm allows for gaps, the majority of type I clusters are short both in terms of the number of cluster members and the number of genes that fall within the boundaries of a cluster (i.e. cluster span is short). The squares on the plot are colour coded according to the number of clusters with a certain gene number and span detected. Darker, more red squares indicate a large number of clusters, whereas paler blue squares indicate a smaller number of clusters.
